# Influence of menstrual cycle phase on inflammatory and vascular responses to acute passive heating in healthy young women

**DOI:** 10.1113/EP092680

**Published:** 2025-10-13

**Authors:** Yunuo Su, Emma O'Donnell, Stephen J. Bailey, Christof A. Leicht

**Affiliations:** ^1^ School of Sport, Exercise and Health Sciences Loughborough University Loughborough UK; ^2^ Peter Harrison Centre for Disability Sport Loughborough University Loughborough UK

**Keywords:** female physiology, heat therapy, inflammation

## Abstract

The aim of this study was to determine whether inflammatory and vascular responses to passive heating differ between the early follicular phase (EFP) and the mid‐luteal phase (MLP) of the menstrual cycle. Ten healthy, naturally menstruating females (26 ± 3 years of age; body mass index 21.4 ± 1.9 kg/m^2^) were assessed during EFP and MLP. Participants underwent 60 min whole‐body passive heat exposure (71°C ± 2°C, dry heat) in both phases. Outcomes included body temperature, interleukin‐6, interleukin‐1 receptor antagonist and plasma nitrite concentrations, cutaneous vascular conductance, blood pressure, arterial stiffness and perceptual responses. Rectal temperature and mean skin temperature increased during heat exposure but did not differ between EFP and MLP. Likewise, heat exposure increased interleukin‐6, interleukin‐1 receptor antagonist and plasma nitrite concentrations, with no differences between menstrual cycle phases. However, brachial (EFP, 75 ± 4 mmHg vs MLP, 72 ± 5 mmHg; *p* = 0.040) and central (EFP, 75 ± 4 mmHg vs MLP, 72 ± 5 mmHg; *p* = 0.042) mean arterial pressures were higher in EFP than in MLP at 40 min of heat exposure. Additionally, arterial stiffness declined more in EFP (−13% ± 7%) than in MLP (−5% ± 6%; *p* = 0.019) from the end of heat exposure to 30 min into recovery. Perceptual responses, including thermal sensation and comfort, were similar between menstrual cycle phases, but skin wetness perception was heightened during EFP. In conclusion, the inflammatory and plasma nitrite responses to passive heating did not differ between EFP and MLP. However, some vascular function and perception parameters were affected by the menstrual cycle phase.

## Abstract (highlights)

- **What is the central question of this study?**
  We sought to determine whether inflammatory and vascular responses to passive heating differ between the early follicular phase and the mid‐luteal phase of the menstrual cycle.
- **What is the main finding and its importance?**
  There was no difference in body temperature between phases in the studied cohort, and passive heating did not result in differing inflammatory responses between menstrual cycle phases. However, a lower mean arterial pressure was found in the mid‐luteal phase, suggesting that vascular responses to heat stress are affected by the menstrual cycle phase.

## 1 INTRODUCTION

Hyperthermia has been shown to induce an acute interleukin (IL)‐6 response, stimulating an acute anti‐inflammatory response [e.g., via interleukin‐1 receptor antagonist (IL‐1ra)], which is thought to contribute to lower inflammatory resting levels over time (Hoekstra et al., 2020). However, the majority of studies have focused on male participants (Hoekstra et al., 2018, 2021; Kaldur et al., 2016) or have overlooked the menstrual cycle phases in females, which might influence the effects of passive heating (Cheng et al., 2021). Specifically, fluctuations in oestrogen and progesterone, typically low in the early follicular phase but high in the mid‐luteal phase, might impact immune system metabolism (Draper et al., 2018). For example, higher oestradiol concentrations are associated with lower urinary cytokine concentrations of IL‐1β, IL‐6, IL‐8 and RANTES (regulated by activated, normal T‐cell expression and secretion) (Whitcomb et al., 2014). Conversely, during the luteal phase, in peripheral blood mononuclear cells, pro‐inflammatory genes have been shown to be upregulated, with a tendency for downregulation of anti‐inflammatory genes compared with the follicular phase (Northoff et al., 2008). In line with this, IL‐10 concentration has been shown to be lower in the luteal phase compared with the follicular phase following high‐intensity intermittent exercise (Minuzzi et al., 2022). Although we are aware of only a few passive heating research studies that controlled for the menstrual cycle in female participants (Ravanelli et al., 2023; Snipe & Costa, 2018), they investigated only specific menstrual cycle phases and did not make comparisons between phases. Given the conflicting evidence on menstrual cycle‐related differences in inflammatory variables, research into the heating‐induced inflammatory response in different phases of the menstrual cycle is warranted.

The menstrual cycle phase also affects resting core temperature, in addition to core temperature thresholds for sweating and cutaneous vasodilatation, by modulating central thermoregulatory mechanisms (Inoue et al., 2005). For instance, during heat exposure, core body temperature and skin blood flow are usually higher in the luteal phase compared with the follicular phase (Inoue et al., 2005; Lee et al., 2014). This can again be explained, at least in part, by hormonal changes, with progesterone leading to an increase in body temperature (Charkoudian & Johnson, 2000) and blood flow (Harrison et al., 2006). Both progesterone and oestrogen influence autonomic thermoregulatory processes, demonstrated by increased thresholds for greater cutaneous vasodilatation during the luteal phase (Charkoudian et al., 1999). Furthermore, oestrogen affects endothelial function through increased nitric oxide (NO) production, decreasing vascular tone (Chambliss & Shaul, 2002). In accordance with this, higher oestrogen concentrations during the menstrual cycle are associated with reduced peripheral blood pressure and arterial stiffness (Adkisson et al., 2010; Dubey, 2002). In contrast, low oestrogen concentrations, as found in postmenopausal women, decrease NO production, which is associated with increased peripheral resistance and arterial stiffness, contributing to higher blood pressure (Zanchetti et al., 2005). It has been shown that plasma nitrite, a proxy marker for circulating endothelium‐derived nitric oxide (Lauer et al., 2001), is elevated following passive heating (Hoekstra et al., 2018; Su et al., 2025). However, again, it is not currently known whether this response might be modulated by the fluctuations in oestrogen and progesterone concentrations throughout the menstrual cycle.

Thermal perception is a key concern when designing passive heating protocols, because exposure to high temperatures can lead to discomfort (Hoekstra et al., 2018), which might potentially be affected by body temperature fluctuations across the menstrual cycle. The influence of menstrual cycle phase on thermal comfort has been investigated previously, but in the context of mild cold exposure, in which no differences in thermal sensation or thermal comfort were found (Matsuda‐Nakamura et al., 2015). However, it remains to be determined whether the menstrual cycle phase impacts on thermal sensation and comfort during whole‐body heat exposure that substantially increases body temperature.

Theref aim of the present study was to investigate potential differences in inflammatory and vascular function responses to passive heat stress during the early follicular phase (EFP) and mid‐luteal phase (MLP) of the menstrual cycle. We hypothesized that: (1) owing to the higher body temperature normally found in the MLP, IL‐6 would increase more in the MLP than in the EFP, resulting in a more pronounced increase in IL‐10 and IL‐1ra; (2) plasma nitrite concentration and skin perfusion during hyperthermia would be higher in MLP compared to EFP; and (3) blood pressure and vascular stiffness would be lower in MLP than in EFP.

## 2 MATERIALS AND METHODS

### 2.1 Ethical approval

Participants were given oral and written information about the procedures and the possible risks of participating in the study, after which they provided written informed consent. The study was approved by the Loughborough University Ethics Committee (project code: 12888), adhering to the ethical standards outlined in the *Declaration of Helsinki*, except for registering in a public database.

### 2.2 Participants

This study investigated 11 healthy females. For exploratory analyses, participants were stratified into a high‐progesterone cohort, with luteal phase progesterone concentrations >3 ng/mL, and a low‐progesterone cohort, with luteal phase progesterone values of <3 ng/mL (Wathen et al., 1984), which led to the exclusion of one female, because she presented with progesterone concentrations consistent with the high‐progesterone cohort, but with oestradiol concentrations in the range of the low‐progesterone cohort. Consequently, 10 healthy females (two European and eight East Asian; age, 26 ± 3 years; body mass, 59.7 ± 5.6 kg; height, 1.7 ± 0.1 m; body mass index, 21.4 ± 1.9 kg/m^2^; self‐reported structured exercise, 5.3 ± 2.3 h/week; high‐progesterone cohort, *n* = 5; low‐progesterone cohort, *n* = 5) were analysed. All participants were non‐smokers and normotensive [<120 mmHg systolic blood pressure (SBP) and <80 mmHg diastolic blood pressure (DBP)]; none was taking medication at the time of the study, and none had a history of cardiovascular or inflammatory complications. All participants had a natural menstrual cycle (28–35 days; self‐reported), none used hormone‐based contraception. All participants had lived in the UK for ≥1 year prior to taking part in the study.

### 2.3 Experimental design

Participants attended baseline anthropometric measurements in a preliminary visit; body composition was assessed using a seca mBCA 515 (Hamburg, Germany). EFP was defined as days 1–7 of the menstrual cycle; the typical menstrual cycle length self‐reported by participants was used to determine MLP (the 4 day period in the middle between ovulation and the predicted first day of the next menstrual cycle). Ovulation was confirmed by a positive result using ovulation test strips (One Step, Hangzhou ALLTEST Biotech Co., Ltd, Hangzhou, China; sensitivity: 20 mIU/L) for all participants.

Laboratory testing was conducted on two main occasions in random order during EFP and MLP. Prior to the first visit, dietary intake and exercise were standardized by recording 24 h of food consumption. Participants replicated this for the subsequent main trial, refraining from engaging in physical exercise and from consuming caffeine and alcohol in this period. Sessions commenced between 11.00 and 13.00 h to minimize the impact of circadian variations on outcome measures.

### 2.4 Experimental procedures

**FIGURE 1:**
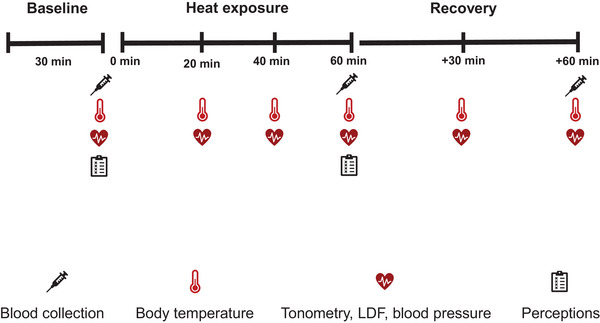
Schematic representation of experimental procedure. Facial cooling was applied during the final 20 min of heat exposure. Blood samples were obtained to measure sex hormones, inflammatory markers and plasma nitrite. Body temperature measurements were rectal and skin temperatures. The perceptions assessed were thermal sensation, thermal comfort and skin wetness. Abbreviation: LDF, laser Doppler flowmetry.

The experimental protocol was identical for EFP and MLP and consisted of 60 min of passive heat exposure (Figure 1). Heating of the whole body below the neck was applied using a Cocoon POD heating device (Wellness USA, Minneapolis, MN, USA), using the ‘high‐hyper’ setting, including infrared heating (EFP, 71.4°C ± 2.0°C and 6.4% ± 2.6% humidity; MLP, 71.4°C ± 2.0°C and 6.5% ± 3.1% humidity). The temperature and humidity in the Cocoon POD were measured using an iButton (Maxim, San Jose, CA, USA) attached to the roof of the device, at the chest level of the participants. To improve comfort and ensure the completion of the intervention, facial cooling was applied during the final 20 min of the heating phase; two circular fans, 10 cm in diameter, located on either side of the face, produced an airflow of 4 m/s, as measured by a Kestrel 3000 Environmental Meter (Richard Paul Russell Ltd, Lymington, UK). Participants were permitted to consume water ad libitum during the sessions, with no significant difference in water intake between EFP (210 ± 144 mL) and MLP (188 ± 166 mL, *p* = 0.793). To mitigate any potential variations in body temperature, thermal perception or heat exposure as a result of clothing, participants wore a standard T‐shirt and shorts made from 100% polyester for both main trials. Ambient laboratory conditions were controlled and did not differ between visits (EFP, 21.6°C ± 0.7°C and 41.5% ± 13.2% relative humidity; MLP, 21.9°C ± 0.9°C and 40.9% ± 15.5%; *p* ≥ 0.322), measured using a wireless weather station (OP‐WS01/WH1170, Opes).

Participants completed the skin preparation for the laser Doppler flowmetry (LDF; moorVMS‐LDF, Moor Instruments, Axminster, UK) measurement themselves by shaving the relevant areas 24 h prior to the test. On arrival, participants inserted a rectal temperature probe 10 cm beyond the anal sphincter for the measurement of rectal temperature (*T*_rec_). Nude body mass was then assessed to the nearest 0.05 kg (Seca 284, Hamburg, Germany). Skin temperature sensors (iButtons) were fitted on the chest, arm, thigh and calf using Transpore™ surgical tape (3M Healthcare, St Paul, MN, USA). Measurements for skin temperature (*T*_skin_) were taken from the left side of the body, and the mean *T*_skin_ was calculated using the following formula: Mean *T*_skin_ = 0.3 × chest *T*_skin_ + 0.3 × arm *T*_skin_ + 0.2 × thigh *T*_skin_ + 0.2 × calf *T*_skin_ (Ramanathan, 1964).

Once fully instrumented, participants rested supine for 30 min at room temperature. During this rest period, the skin of the left forearm and left thigh was cleansed with distilled water, then the LDF sensors were applied to the forearm and thigh. At the end of the rest period, the temperature measures, heart rate (HR; Polar, Kempele, Finland), blood pressure (Omron Healthcare, Kyoto, Japan) and vascular function (pulse wave analysis) metrics were recorded. Furthermore, participants reported their thermal sensation (Epstein & Moran, 2006), thermal comfort (Gagge & Nishi, 1976) and skin wetness (Filingeri et al., 2015). Blood samples were collected via venepuncture of an arm vein after completing the physiological and perceptual measurements.

After the rest period, participants entered the Cocoon POD for passive heating. Physiological and perceptual measurements were assessed every 20 min during the session. At the end of the passive heating period, blood samples were collected immediately. After that, nude body was reassessed to estimate the amount of sweat loss, taking into account water intake. After the session, participants rested supine at room temperature for an additional 60 min to assess physiological and perceptual responses at 30 and 60 min postintervention, followed by another blood sample collection.

### 2.5 Measurements

#### 2.5.1 Pulse wave analysis

Pulse wave analysis was assessed at the radial artery using applanation tonometry methods (SphygmoCor; Atcor Medical, Sydney, NSW, Australia) to determine central blood pressure and indices of arterial stiffness. Radial artery pressure waveforms, calibrated against brachial artery blood pressure readings, were used to derive the central aortic pressure waveform using a validated generalized transfer function (Chen et al., 1997). Pulse wave analysis indices of interest included aortic blood pressure components [central systolic (SBP), diastolic (DBP), mean arterial (MAP) and pulse pressure (PP)], and the augmentation index adjusted for heart rate of 75 beats/min (AIx@HR75) is reported.

Baseline measurements were taken after 20 min of supine rest. The 20 and 40 min measurements were taken between 15 and 20 min and between 35 and 40 min of the intervention, respectively. Postintervention and 60 min recovery measurements were taken between 55 and 60 min of the intervention and between 55 and 60 min of recovery, respectively. All measurements and analyses were conducted by the same operator (Y.S.), with each measurement performed in duplicate. The two readings were averaged, and only datasets achieving an operator index of ≥80% were included in this study.

#### 2.5.2 Laser Doppler flowmetry

The LDF probes were placed on the mid‐ventral forearm and mid‐ventral thigh, flush with the skin, to measure red blood cell flux, an index of skin perfusion (Nilsson et al., 1980). All participants were instructed to remain as still as possible for 5 min before data recording. The raw LDF data recorded at 40 Hz were then averaged per second, and data points that deviated by >3SD from the average were excluded from analysis. Skin perfusion data were recorded at baseline, at 20, 40 and 60 min during the intervention and at 30 and 60 min during the recovery period, and are reported as 30 s averages. All flux values were converted to cutaneous vascular conductance (CVC) using the formula: CVC = flux/brachial mean arterial pressure.

#### 2.5.3 Blood analyses

Blood samples were collected using K_3_EDTA and lithium–heparin monovettes and centrifuged at 2,360 *g* for 10 min at 4°C. The resulting plasma was aliquoted and stored at −80°C until batch analysis.

K_3_EDTA plasma was used to analyse concentrations of 17β‐estradiol (ALPCO, Salem, NH, USA), progesterone (Abcam, Cambridge, UK), IL‐6 (High Sensitivity, R&D Systems, Abingdon, UK), IL‐1ra (Cytoscreen, Invitrogen, Paisley, UK) and IL‐10 (High Sensitivity, Invitrogen, Paysley, UK) using ELISAs with intra‐plate CVs of 2.5%, 2.4%, 6.0%, 7.0% and 2.5%, respectively.

Lithium–heparin plasma nitrite concentrations were determined by ozone chemiluminescence (Bailey et al., 2009). Initially, 500 µL of heparinized plasma was deproteinized by mixing with an equal volume of ice‐cold ethanol and centrifuged at 2,360 *g* for 10 min. The supernatant was then introduced into a gas‐tight purge vessel via 200 µL injection through a septum. Nitric oxide (NO) was subsequently produced by reducing plasma nitrite in the presence of glacial acetic acid and 4% w/v aqueous sodium iodide solution. The chemiluminescence signal of NO reacting with ozone was recorded using a Sievers NOA 280i analyser (Analytix, Durham, UK), with the millivolt signal area being converted to concentration using a standard curve from nitrite standards across the nanomolar concentration range.

Haematocrit and haemoglobin concentrations were measured in duplicate using a microcentrifuge and a Yumizen H500 automatic analyser (Horiba Medical, Montpellier, France). These measurements were used to adjust the concentrations of IL‐6, IL‐1ra, IL‐10 and nitrite for changes in plasma volume (Dill & Costill, 1974).

### 2.6 Statistical analysis

The sample size was determined a priori based on anticipated changes in *T*_rec_ across menstrual cycle phases, drawing on data reported by Stone et al. (2021). In that study, an effect size of 1.5 was observed for the difference in rectal temperature between the mid‐luteal and early follicular phases. Using this estimate, and assuming a significance level (α) of 0.05 and a statistical power (1 − β) of 0.99, the minimum required sample size was calculated to be *n* = 9 for a repeated‐measures design.

All statistical procedures were performed using the 29^th^ version of SPSS (IBM Corp., Armonk, NY, USA). Data are presented as the mean ± SD. Normality and sphericity were checked using Shapiro–Wilk and Mauchley's tests, respectively. Changes in physiological and thermoregulatory parameters, in addition to 17β‐estradiol, progesterone, IL‐6, IL‐1ra, IL‐10 and nitrite concentrations, were analysed using a three‐way repeated‐measures ANOVA (phase × time × progesterone cohort), checking pairwise comparisons when significant interactions were detected. A Bonferroni correction was applied to control for multiple comparisons in *post hoc* tests. Sex hormone concentrations were compared between racial groups using a Mann–Whitney *U*‐test. Changes in perceptual measures were analysed non‐parametrically. The Friedman test was used to test for the effect of time in each condition, whereas Wilcoxon signed‐rank tests were used for pairwise comparisons. Mann–Whitney *U*‐tests were performed to assess phase differences in perception scale data. Finally, Spearman's 𝑟 was used to compute correlations, examining the bivariate relationships between baseline progesterone and oestradiol concentrations and *T*_rec_, blood pressure and thermal perception measured at the end of heat exposure as well as resting *T_rec_*. The level of significance was set at *p* < 0.05.

## 3 RESULTS

### 3.1 Hormone concentrations

**FIGURE 2:**
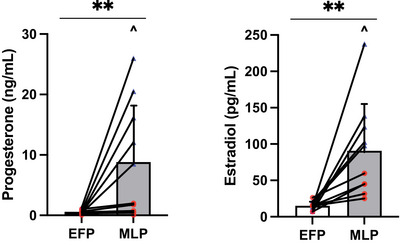
Plasma concentrations of progesterone and oestradiol in the early follicular phase (EFP) and mid‐luteal phase (MLP), the high‐progesterone cohort (triangles) and low‐progesterone cohort (circles), respectively. ^**^Different between the two phases in the high‐progesterone cohort. ^^^Different between high‐ and low‐progesterone cohorts (*p* < 0.05).

Main effects of phase, progesterone cohort and a phase × progesterone cohort interaction were observed in progesterone and oestradiol concentrations (*p* ≤ 0.006). Progesterone and oestradiol concentrations in EFP were lower than in MLP in the high‐progesterone cohort (*p* < 0.001), but not in the low‐progesterone cohort (*p* ≥ 0.217). Progesterone and oestradiol concentrations were higher in the high‐progesterone cohort than in the low‐progesterone cohort in MLP (*p* ≤ 0.005). The progesterone and oestradiol concentrations of the cohorts did not differ in EFP (*p* ≥ 0.642; Figure 2). There were no differences between European and East Asian participants in progesterone or oestradiol concentrations during EFP or MLP (*p* ≥ 0.68).

### 3.2 Body temperature

**FIGURE 3:**
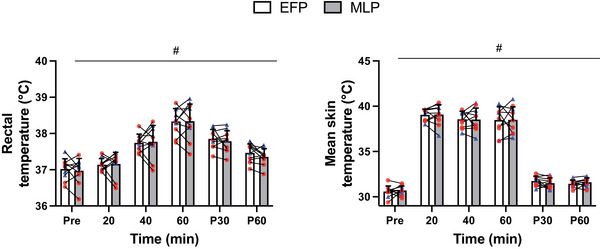
Rectal and mean skin temperature response to heat exposure in the early follicular phase (EFP) and mid‐luteal phase (MLP), the high‐progesterone cohort (triangles) and low‐progesterone cohort (circles), respectively. ^#^Effect of time (*p* < 0.05).

The *T*_rec_ increased following heat exposure (*p* < 0.001), with no effect of phase (*p* = 0.746; phase × time *p* = 0.412) or progesterone cohort (*p* = 0.080; Figure 3). Likewise, there was a main effect of time for mean *T*_skin_ (*p* < 0.001), with no effect of phase (*p* = 0.922; phase × time *p* = 0.813) or progesterone cohort (*p* = 0.610).

### 3.3 Inflammatory and nitrite response

**FIGURE 4:**
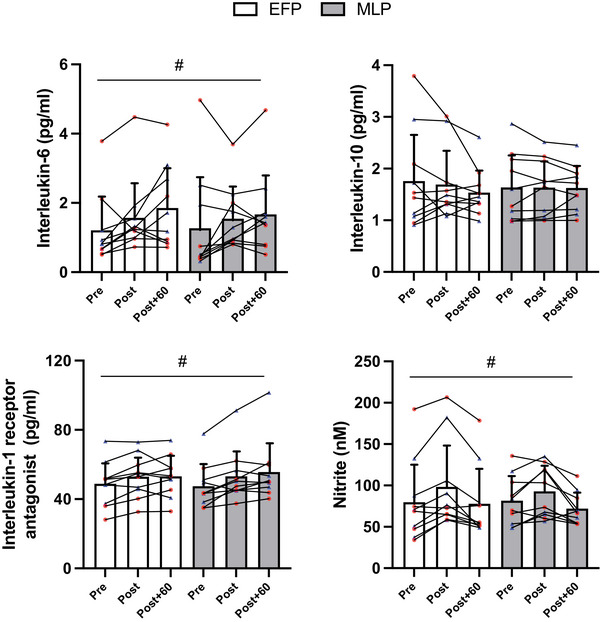
Inflammatory and plasma nitrite responses to heat exposure in the early follicular phase (EFP) and mid‐luteal phase (MLP), the high‐progesterone cohort (triangles) and low‐progesterone cohort (circles), respectively. ^#^Effect of time (*p* < 0.05).

The concentrations of IL‐6, IL‐1ra and plasma nitrite increased after heat exposure, with no phase difference (phase, *p* ≥ 0.801; time, *p* ≤ 0.016; phase × time, *p* ≥ 0.237), and were unaffected by progesterone cohort (*p* ≥ 0.322; Figure 3). Interleukin‐10 was not affected by heat exposure (*p* = 0.460), phase (*p* = 0.810; phase × time, *p* = 0.362) and progesterone cohort (*p* = 0.668; Figure 4).

### 3.4 Cutaneous vascular conductance

**FIGURE 5:**
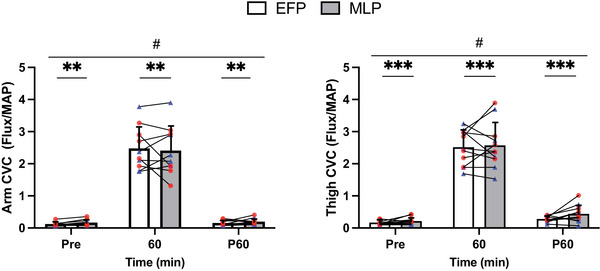
Cutaneous vascular conductance (CVC) to heat exposure in the early follicular phase (EFP) and mid‐luteal phase (MLP), the high‐progesterone cohort (triangles) and low‐progesterone cohort (circles). ^#^Effect of time. ^**^Different between the two phases in the high‐progesterone cohort. ^***^Different between the two phases in the low‐progesterone cohort (*p* < 0.05).

The CVC in the arm and thigh increased following heat exposure (*p* < 0.001). For arm CVC, no main effect of phase was observed (phase, *p* = 0.501; phase × time, *p* = 0.948; Figure 5). However, a significant phase × progesterone cohort interaction was found (*p* = 0.023), with higher arm CVC during MLP compared with EFP in the high‐progesterone cohort (*p* = 0.031); this effect was not observed in the low‐progesterone cohort (*p* = 0.199).

In the thigh, no significant main effect of phase was found (phase, *p* = 0.084; phase × time, *p* = 0.623), but a significant phase × progesterone cohort interaction emerged (*p* = 0.036), with higher thigh CVC during MLP than during EFP in the low‐progesterone cohort (*p* = 0.016), but not in the high‐progesterone cohort (*p* = 0.713).

### 3.5 Blood pressure and pulse wave analysis

**FIGURE 6:**
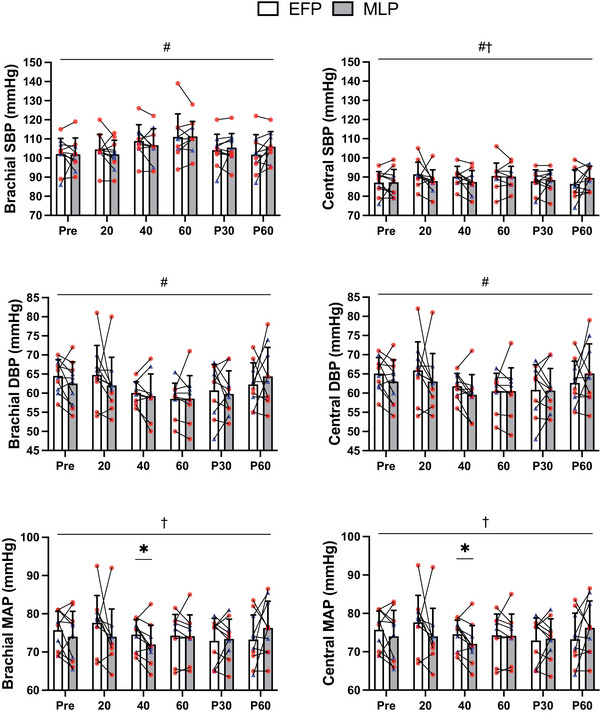
Brachial and central blood pressure responses to heat exposure in the early follicular phase (EFP) and mid‐luteal phase (MLP), the high‐progesterone cohort (triangles) and low‐progesterone cohort (circles), respectively. Abbreviations: DBP, diastolic blood pressure; MAP, mean arterial pressure; SBP, systolic blood pressure. ^#^Effect of time. ^†^Phase × time interaction. ^*^Different between EFP and MLP (*p* < 0.05).

Brachial SBP increased after heat exposure (*p* < 0.001), with no difference between phases (phase, *p* = 0.931; phase × time, *p* = 0.124; Figure 6). Brachial DBP decreased after heat exposure (*p* < 0.001) without phase differences (phase, *p* = 0.604; phase × time, *p* = 0.285). Brachial MAP showed a phase × time interaction (*p* = 0.018), with MAP in EFP being higher than MLP at 40 min of heat exposure (*p* = 0.040).

There was no main effect of phase for central SBP (*p* = 0.759), but there were a main effect of time (*p* = 0.042) and a phase × time interaction (*p* = 0.047). Central DBP decreased (*p* < 0.001) without phase differences (phase, *p* = 0.614; phase × time, *p* = 0.161). Central MAP showed a phase × time interaction (*p* = 0.020), with higher MAP in EFP compared with MLP at 40 min of heat exposure (*p* = 0.042).

No effect of progesterone cohort was found for brachial and central SBP, DBP or MAP (*p* ≥ 0.685).

**FIGURE 7:**
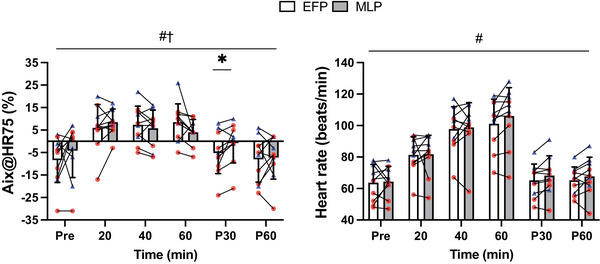
Augmentation index adjusted for a heart rate of 75 beats/min (AIx@HR75) and heart rate responses to heat exposure in the early follicular phase (EFP) and mid‐luteal phase (MLP), the high‐progesterone cohort (triangles) and low‐progesterone cohort (circles), respectively. ^#^Effect of time. ^†^Phase × time interaction. ^*^Different between EFP and MLP (*p* < 0.05).

There was a main effect of time (*p* < 0.001) and a phase × time interaction (*p* = 0.036) in Aix@HR75, resulting in a lower Aix@HR75 for EFP than for MLP at 30 min after heat exposure (*p* = 0.019; Figure 7). Aix@HR75 was unaffected by progesterone cohort (*p* = 0.250). Heart rate was increased during heat exposure, with no phase difference (phase, *p* = 0.196; time, *p* < 0.001; phase × time, *p* = 0.563).

### 3.6 Perceptual response and sweat loss

Thermal sensation and thermal comfort were affected by heat exposure in both phases (*p* < 0.001), with no phase (*p* ≥ 0.317) or progesterone cohort difference (*p* ≥ 0.166; Table 1). Skin wetness was increased after heat exposure in both phases (*p* < 0.001). Skin wetness in EFP was higher than in MLP at 60 min of heat exposure (*p* = 0.046) but was not affected by progesterone cohort (*p* ≥ 0.419). Sweat loss to heat exposure was not different between EFP and MLP (phase, *p* = 0775; phase × progesterone cohort, *p* = 0.314) and was not affected by progesterone cohort (*p* = 0.343).

**TABLE 1:** Thermal perception and sweat loss in response to passive heat exposure.

Time point	Thermal sensation (1–9)		Thermal comfort (0–4)		Skin wetness (−3 to +3)		Sweat loss (mL)	
	EFP^†^	MLP^†^	EFP^†^	MLP^†^	EFP^†^	MLP^†^	EFP	MLP
Pre	4.1 ± 1.0	4.3 ± 0.7	0.1 ± 0.3	0.0 ± 0.0	−0.5 ± 0.8	−0.4 ± 0.7	–	–
Post	8.6 ± 0.5	8.6 ± 0.7	2.7 ± 1.3	2.7 ± 1.3	**2.7 ± 0.7** ^*^	**2.4 ± 0.7** ^*^	636 ± 143	648 ± 143

*Note*: All data are presented as mean ± SD. Significant differences are highlighted in bold. Thermal sensation ranged from 1, very cold to 9, very hot. Thermal comfort ranged from 0, comfortable to +4, very uncomfortable. Skin wetness ranged from −3, very dry to +3, very wet. Abbreviations: EFP, early follicular phase; MLP, mid‐luteal phase.

* Significantly different between EFP and MLP (*p* < 0.05).

^†^ Significantly different between Pre and Post (*p* < 0.05).

### 3.7 Correlations

In EFP, resting *T*_rec_ was negatively correlated with oestradiol concentration (*r *= −0.737, *p* = 0.016; Figure S1). Oestradiol concentrations were inversely correlated with both brachial MAP (*r* = −0.650, *p* = 0.042) and central MAP (*r* = −0.650, *p* = 0.042) at the end of heat exposure. In addition, oestradiol concentration was positively correlated with thermal comfort (*r* = 0.654, *p* = 0.040) at the end of heat exposure.

In MLP, resting *T*_rec_ was positively correlated with progesterone concentration (*r* = 0.770, *p* = 0.009). A positive correlation was also found between peak *T*_rec_ and oestradiol concentration (*r* = 0.636, *p* = 0.048).

## 4 DISCUSSION

We sought to explore whether EFP and MLP of the menstrual cycle would affect the inflammatory response and vascular function in response to passive heating. We found that: (1) heat exposure increased plasma IL‐6, IL‐1ra and nitrite concentrations to the same extent in both menstrual cycle phases; (2) increases in arm skin perfusion were unaffected by menstrual cycle phase but were affected by progesterone concentration in MLP; (3) MAP at 40 min of heat exposure was higher in EFP compared with MLP; and (4) the progesterone concentration in MLP in 5 of the 10 analysed participants was below the MLP reference value of 3 ng/mL.

In the present study, the MLP trial was scheduled according to calendar tracking, and ovulation was confirmed by a surge in urine luteinizing hormone. However, our results show that a positive ovulation test does not accurately predict rises in progesterone. The reasons for this are unclear but might be attributable, in part, to the known common presentation of menstrual cycle disturbances in recreationally active women, such as luteal phase deficiency which, despite presence of ovulation, can result in progesterone insufficiency (De Souza et al., 1998). Studies demonstrate that among recreationally active women exercising for 5.0 ± 0.7 h/week, similar to that observed in the present study (5.3 ± 2.3 h/week), a sample prevalence of 43% for luteal phase defect was detected in 66 menstrual cycles (De Souza et al., 1998). Anovulatory cycles were also detected at a sample prevalence of 12%. These findings emphasize that eumenorrheic menstrual cycles commonly present with ovulatory or luteal dysfunction, which might help to explain the lower than expected progesterone concentrations observed in some participants in this study.

### 4.1 Body temperature

The *T*_rec_ and *T*_skin_ increased during passive heating across both menstrual cycle phases. However, *T*_rec_ and *T*_skin_ did not differ between EFP and MLP at rest and during passive heating in the whole cohort. This contradicts previous research, which found *T*_rec_ to be higher in the luteal phase than in the follicular phase at rest and after passive heating (Inoue et al., 2005; Kuwahara et al., 2005). Progesterone contributes to the initiation of cutaneous vasodilatation and sweating through its modulation of central thermoregulatory pathways, particularly within the hypothalamic preoptic area (Charkoudian & Johnson, 2000; Charkoudian et al., 1999). This mechanism might help to explain the luteal phase‐related increase in body temperature. Indeed, we found a positive correlation between progesterone concentration and resting *T*_rec_ in the present study. However, in the whole cohort, despite higher concentrations of progesterone observed in MLP (8.8 ± 9.5 ng/mL) compared with EFP (0.6 ± 0.4 ng/mL), *T*_rec_ measured at rest and during heating was not higher in MLP when compared with EFP. This aligns with findings by Zheng et al. (2022), who reported no differences in *T*_rec_ between the luteal and follicular phases in females with regular menstrual cycles (21–35 days), whether at rest or during cycling tasks in dry, warm conditions (Zheng et al., 2022). Both the study by Zheng et al. (2022) and the present study included females with low progesterone concentrations in the luteal phase, which might explain the lack of difference in *T*_rec_ between EFP and MLP.

We found a negative correlation between oestradiol and resting *T*_rec_, but a positive correlation between oestradiol and peak *T*_rec_ during passive heating. This is in line with Zheng et al. (2022), who showed positive correlations between oestradiol concentrations and peak *T*_rec_ during cycling in dry, warm conditions. However, it seemingly contrasts with previous findings, because oestradiol is generally associated with vasodilatation, enhanced heat dissipation and reduced body temperature (Charkoudian & Stachenfeld, 2014; Lei et al., 2019). The discrepancy is likely to lie in the difference in ambient conditions between experiments. At room temperature, oestradiol‐induced vasodilatation promotes heat loss and lowers core temperatures (Grucza et al., 1993), whereas during heat exposure, when heat gain exceeds heat loss, dilated vessels might, in fact, facilitate heat transfer from the environment to body fluids and muscles (Cheung et al., 2000; Heinonen et al., 2011), resulting in an elevated *T*_rec_ when the evaporative cooling potential is insufficient. In the present study, the capsule‐like structure of the Cocoon POD heating device restricts airflow, limiting heat dissipation via evaporation. Combined with oestradiol‐induced vasodilatation, this reduction in evaporative cooling capacity might increase *T*_rec_, which is reflected in our finding of the positive correlation between oestradiol concentration and peak *T*_rec_.

### 4.2 Inflammatory response

Elevated progesterone levels contribute to the higher body temperature in the luteal phase, which typically rises by 0.3°C–0.7°C compared with the follicular phase (Baker et al., 2020). Even small fluctuations in core body temperature (0.5°C increase) have the potential to induce an acute inflammatory response (Su et al., 2025), which led to the formulation of our hypothesis of a differential inflammatory response across EFP and MLP. However, no difference in the IL‐6 and IL‐1ra increase was observed between EFP and MLP. Given the influence of core temperature on IL‐6 upregulation (Brunt et al., 2018; Hoekstra et al., 2020), this might be attributable to the absence of differences in *T*_rec_ across the two phases. Our results are in line with evidence reported for a cycling exercise task, which showed IL‐6 increasing, with no differences between the follicular and luteal phases, and no differences in *T*_rec_ between the two phases (Zheng et al., 2021). In the present study, we did not find an impact of oestradiol or progesterone on IL‐6 concentrations, as opposed to the report by Whitcomb et al. (2014), which might be related to the larger number of time periods analysed across the menstrual cycle previously (eight), compared with the comparison of only two phases in the present study.

Interleukin‐6 upregulation can stimulate the production of anti‐inflammatory cytokines, such as IL‐1ra and IL‐10 (Petersen & Pedersen, 2005). Therefore, the similar acute increase in IL‐1ra between EFP and MLP could be attributed to the similar IL‐6 response between phases. Notably, IL‐10 did not increase significantly in response to the IL‐6 elevation. Although IL‐6 was elevated by heat exposure, it might not have reached the threshold necessary for substantial IL‐10 upregulation. For instance, acute total body resistance exercise elevated IL‐6 from 1.6 to 3.4 pg/mL without a significant IL‐10 response (Benini et al., 2015). In contrast, a marathon, which induces more severe inflammatory responses (IL‐6 increase by ∼20 pg/mL), can induce a pronounced increase in IL‐10 (Gill et al., 2015). In the present study, the modest IL‐6 increase by ∼0.7 pg/mL might explain the lack of IL‐10 upregulation.

### 4.3 Vascular response

Elevated plasma nitrite concentrations induce an acute vascular response aimed at enhancing cutaneous blood flow, thereby aiding thermoregulation (Charkoudian, 2003; Johnson & Kellogg, 2010). Circulating nitrite concentration increases with follicular development, mediated by17β‐oestradiol, which increases NO production and bioavailability via the PI3K/Akt pathway (Dimmeler et al., 1999; Rosselli et al., 1994). In the present study, despite higher concentrations of oestradiol in MLP than in EFP, there were no differences in plasma nitrite concentrations between EFP and MLP at baseline and after heat exposure. Furthermore, plasma nitrite concentration did not differ in MLP between the high‐ and low‐progesterone cohorts, despite lower oestradiol concentrations in the low‐progesterone cohort. These results are in line with a previous study that showed no difference in basal plasma nitrate and nitrite concentrations between the early follicular and early and late luteal phases (Adkisson et al., 2010). It is noteworthy that Adkisson et al. (2010) also showed higher plasma nitrate and nitrite concentrations in the late follicular phase (12–14 days after the onset of menses), which is generally associated with peak oestradiol concentrations days before ovulation (Stricker et al., 2006). It is therefore possible that the oestradiol concentrations in the mid‐luteal phase in the present study were not high enough to mediate a greater NO release compared with the early follicular phase.

Although NO participates in cutaneous vasodilatation caused by increased *T*_skin_ (Kellogg et al., 1998, 1999), in the present study we found a higher arm CVC of the high‐progesterone cohort in MLP compared with EFP, without significant differences in plasma nitrite concentrations between these phases. These findings support the direct vasodilatory action of oestradiol or progesterone, independently of NO. For example, oestradiol promotes the release or increased activity of other vasodilatory compounds, such as prostacyclin and endothelium‐hyperpolarizing factor (Flammer & Lüscher, 2010). Progesterone might also contribute through alternative pathways, including modulation of ion channels in vascular smooth muscle and inhibition of L‐type voltage‐gated calcium channels, thereby reducing calcium influx and promoting vasorelaxation (Murphy & Khalil, 1999). However, given that plasma nitrite was measured from venous rather than arterial samples, we cannot exclude the possibility of localized NO effects within specific segments of the vascular tree.

In conduit and resistance vessels, the upper and lower limbs differ in their haemodynamic profiles, with the arms (e.g., forearm) generally exhibiting greater vasodilatory responses to physiological stimuli, whereas the legs (e.g., calf) show stronger sympathetically mediated vasoconstrictor tone (Proctor & Newcomer, 2006). These differences are thought to result, in part, from chronic exposure of the lower limbs to higher transmural pressures owing to gravitational and hydrostatic effects, which lead to increased vascular wall stiffness (Eiken & Kölegård, 2004). In the present study, thigh CVC in the low‐progesterone cohort was higher during MLP compared with EFP. Although greater arterial stiffness in the lower limbs might be expected to attenuate vasodilatory capacity in the skin, the higher oestradiol concentrations observed in MLP (41.3 ± 13.4 pg/mL) relative to EFP (15.8 ± 6.9 pg/mL), despite not reaching statistical significance, might have contributed to enhanced endothelial function and shear stress‐induced vasodilatation sufficient to overcome baseline vascular tone (Forstermann & Sessa, 2012).

The present study found heat exposure to increase plasma nitrite and to decrease both brachial and central DBP during both menstrual cycle phases. This reduction in DBP is likely to be attributable to NO‐mediated vasodilatation, triggered by elevated skin temperature activating endothelial nitric oxide synthase, which reduces systemic vascular resistance (Kellogg et al., 1998). The reduction in brachial and central MAP during heating was more pronounced in MLP than in EFP. This difference, although small in magnitude (3 mmHg), might again be attributed to the vasodilatory effects of oestradiol, with higher concentrations during MLP, reducing systemic vascular resistance and enhancing blood flow, and thereby lowering MAP (Dubey, 2002). The negative correlation between oestradiol concentration and brachial and central arterial MAP in EFP found in the present study supports this postulate and is in line with a previous study (Wenner et al., 2022). The reduction in MAP might lead to a reduction in cardiovascular strain (DeMers & Wachs, 2019), although we must highlight that cardiac load was not assessed directly in this study.

A previous study reported lower resting Aix@HR75 in the early luteal phase than in the early follicular phase, with temporal changes in blood pressure observed to parallel changes in Aix@HR75 (Adkisson et al., 2010). However, in the present study, neither brachial nor central blood pressure showed differences between EFP and MLP at rest or in the recovery from heat exposure. Aix@HR75 was increased in both menstrual cycle phases during heat exposure; furthermore, there was no difference in Aix@HR75 between EFP and MLP at rest. These findings are consistent with previous findings showing no differences in Aix@HR75 increases between the early luteal and early follicular phases before and after an acute bout of resistance exercise (Augustine et al., 2018).

Notably, Aix@HR75 was lower in EFP than in MLP at 30 min of recovery from heat exposure. This might seem unexpected given the lower oestradiol levels during EFP, but this difference might reflect phase‐dependent differences in the vascular recovery process following heat‐induced vasodilatation. In the present study, both brachial and central MAP decreased more during heating in MLP, indicating a stronger vasodilatory response as oestradiol promotes vasodilatation (Charkoudian & Johnson, 1997). This augmented vasodilatation might have triggered a faster rebound in vascular tone during the recovery period, leading to greater arterial stiffness and thus higher Aix@HR75. Moreover, high oestradiol and progesterone concentrations during MLP might activate the renin–angiotensin–aldosterone system, which can result in increased vascular tone and cardiac load (Ounis‐Skali et al., 2006).

### 4.4 Thermal perception

Thermal sensation and comfort did not differ significantly between menstrual cycle phases. Again, this might be explained by the similar rise in *T*_rec_ and *T*_skin_ between EFP and MLP, which are the main determinants of thermal sensation and thermal comfort (Kato et al., 2001). It has been noted that oestradiol upregulation negatively influences thermal perception during exposure of the hands to heat (∼52°C) in women (Stening et al., 2012). The present study partly supports this finding, because oestradiol concentration was correlated with thermal comfort in EFP. However, we did not find thermal comfort to be greater in MLP, despite higher oestradiol concentrations during this phase.

Skin wetness perception was greater in EFP compared with MLP at the end of heat exposure. Notably, humans do not possess specialized receptors in the skin that detect moisture directly; rather, the brain interprets the sensation of moisture through indirect cues, such as temperature and touch (Filingeri et al., 2014). Although no significant differences in sweat loss were observed in the present study, the heightened perception of skin wetness during EFP might suggest that the perception of heat exposure, influenced by temperature and tactile inputs, is more pronounced in MLP compared with EFP.

### 4.5 Limitations

An unexpected finding of the present study was the large proportion of females with MLP progesterone concentrations below the MLP reference value, despite a participant cohort of young, eumenorrhoeic women, and the definition of MLP as the midpoint between confirmed ovulation and onset of the next menstrual cycle. Notably, many prior studies do not measure or report sex hormone concentrations, instead relying solely on timing within the menstrual cycle to define phases (e.g., Snipe & Costa, 2018; Stoner et al., 2012). Consequently, it is plausible that other studies have observed analogous hormone profiles. As a speculative analysis, we therefore included subgroup analyses of the low‐ versus high‐progesterone cohorts, but we appreciate that the small subgroup sizes resulted in a low statistical power for progesterone cohort comparisons. Future studies should include larger sample sizes to explore such cohorts further. Overall, the present study was originally powered based on rectal temperature differences reported in prior literature; however, we acknowledge that this outcome was not the primary focus of the present analysis. Additionally, in this study we investigated only two phases of the menstrual cycle, the early follicular and mid‐luteal phases. This limits the generalizability of the findings to the entire menstrual cycle, because other phases, such as the late follicular phase or ovulation (associated with the highest oestradiol concentration during the cycle), might elicit different physiological or perceptual responses to heat. It is also possible that the true progesterone peak in MLP was missed in some individuals, because testing was conducted at only one discrete time point, which could also help to explain the large proportion of females with progesterone concentration below the expected levels for MLP. Eumenorrhoea was defined by menstrual cycle lengths ≥21 and ≤35 days, in addition to evidence of a luteinizing hormone surge, and no use of hormonal contraception in the 3 months prior to recruitment (Elliott‐Sale et al., 2021). However, this study relied on self‐reported menstrual cycle length from the month preceding testing, along with only one confirmed luteinizing hormone surge prior to MLP testing, which is a limitation of the study. We must also note that testing was conducted in a randomized order across EFP and MLP but was not restricted to the same cycle for each participant, and intra‐individual variability in ovarian hormone concentrations between cycles might thus have influenced the results. Another limitation is that standardization of skin blood flow to a maximum value was not performed in the present study. This might result in diminished comparability across time points and menstrual cycle phases. Finally, future studies might investigate racial differences, which was not done in the present study.

## 5 CONCLUSION

Inflammatory and plasma nitrite responses to passive heating did not differ between EFP and MLP. However, vascular responses were affected by the menstrual cycle phase and sex hormones. Specifically, MAP was higher during heat exposure in EFP compared with MLP, the decrease in Aix@HR75 after heat exposure was greater in EFP than in MLP, and arm skin perfusion was greater in MLP than in EFP only in those with higher progesterone concentrations during MLP. These findings highlight the role of female sex hormones in modulating vascular function during passive heating, whereas inflammatory and plasma nitrite responses appear unaffected by the menstrual cycle phase.

## AUTHOR CONTRIBUTIONS

Yunuo Su, Christof A. Leicht and Emma O'Donnell contributed to the study conception and design. Yunuo Su conducted the experiments. Yunuo Su and Stephen J. Bailey analysed the data, and Yunuo Su wrote the first draft of the manuscript. All authors provided critical feedback, approved the final version of this manuscript and agree to be accountable for all aspects of the work in ensuring that questions related to the accuracy or integrity of any part of the work are appropriately investigated and resolved. All persons designated as authors qualify for authorship, and all those who qualify for authorship are listed.

## CONFLICT OF INTEREST

None declared.

## Supporting information



Bivariate relationships between baseline progesterone and estradiol concentrations and resting rectal temperature, as well as rectal temperature, blood pressure, and thermal perception at the end of heat exposure.

## Data Availability

The data that support the findings of this study are available from the corresponding author upon reasonable request.
