# Supplementary figures and images for: Influence of menstrual cycle phase on inflammatory and vascular responses to acute passive heating in healthy young women
Source: Exp Physiol. 2025 Oct 13:10.1113/EP092680. Online ahead of print. doi: 10.1113/EP092680 (PMC13394663; doi:10.1113/EP092680)

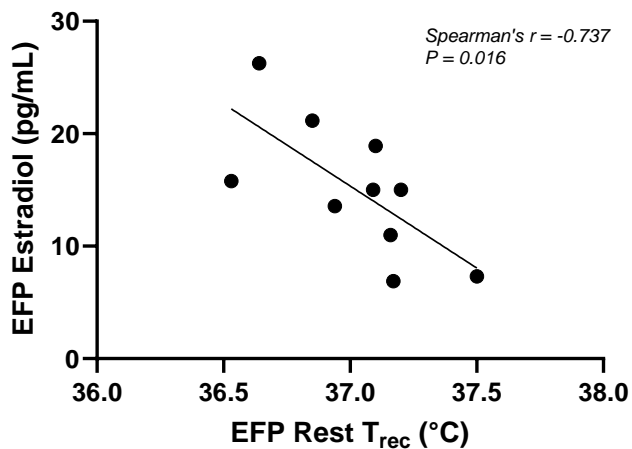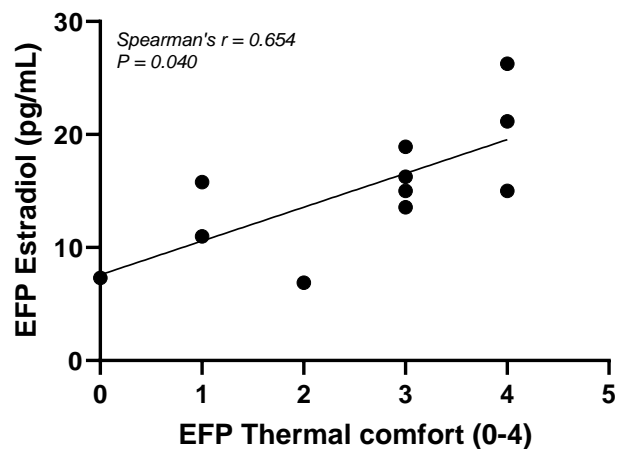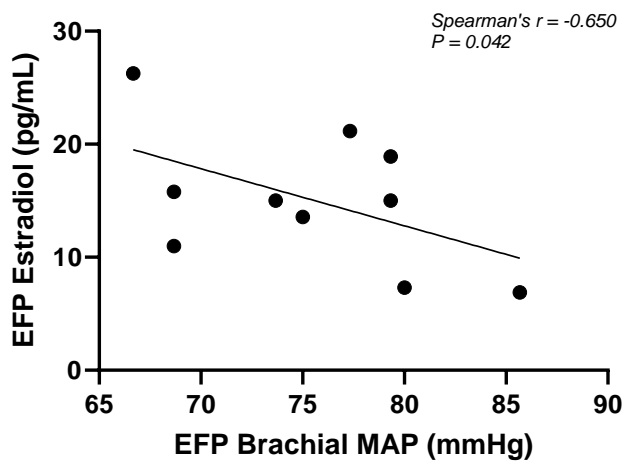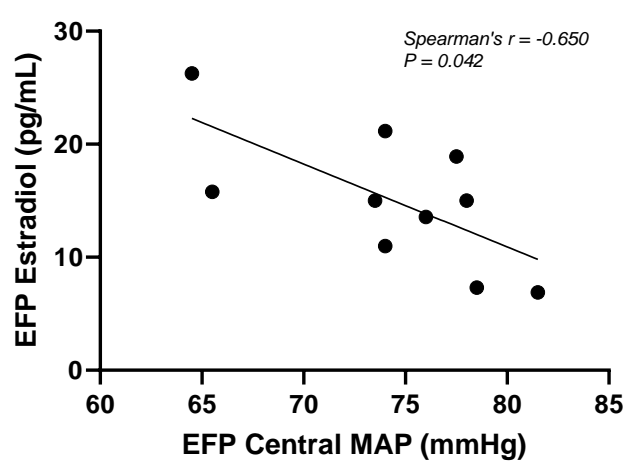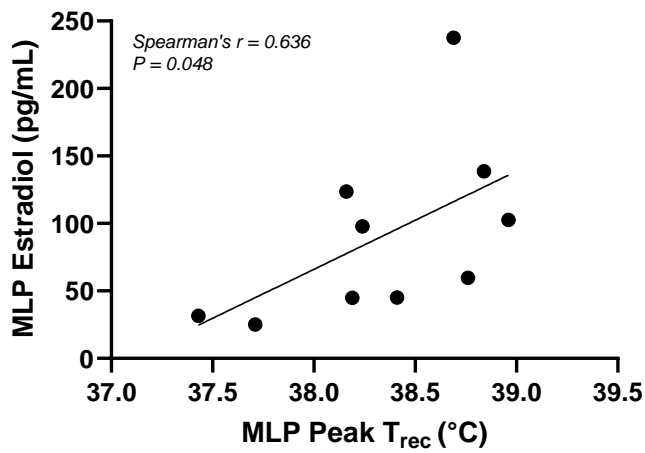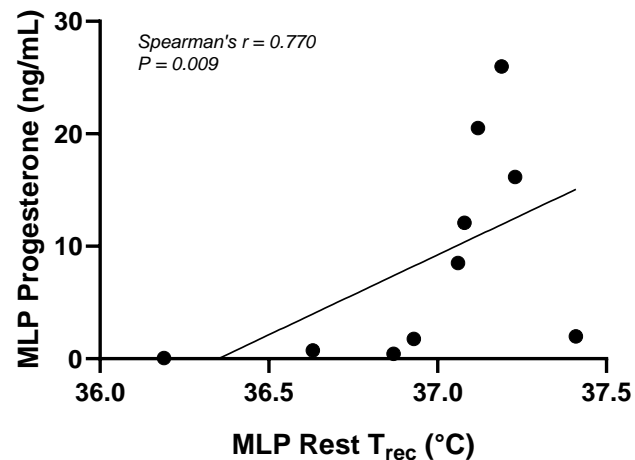

Supplement: Supplementary file 1 — Bivariate relationships between baseline progesterone and estradiol concentrations and resting rectal temperature, as well as rectal temperature, blood pressure, and thermal perception at the end of heat exposure. [file EPH-9999-0-s001.pdf]
